# Farm production diversity and women’s dietary diversity: Evidence from central Tunisia

**DOI:** 10.1371/journal.pone.0263276

**Published:** 2022-02-07

**Authors:** Cédric Gaillard, Eric O. Verger, Sandrine Dury, Marie Claude Dop, Jalila El Ati, on behalf of the MEDINA Study Group

**Affiliations:** 1 CIRAD, UMR MOISA, F-34398 Montpellier, France; 2 MoISA, Univ Montpellier, CIRAD, CIHEAM-IAMM, INRAE, Institut Agro, IRD, Montpellier, France; 3 NUTRIPASS, IRD, Université de Montpellier, SupAgro, Montpellier, France; 4 SURVEN (Nutrition Surveillance and Epidemiology in Tunisia) Research Laboratory, INNTA (National Institute of Nutrition and Food Technology), Tunis, Tunisia; University of Lincoln, UNITED KINGDOM

## Abstract

In the context of studies on the effects of agricultural production diversity, there are debates in the scientific community as to the level of diversification appropriate for improving dietary diversity. In Tunisia, agriculture is a strategic sector for the economy and a critical pillar of its food sovereignty. Using instrumental variable methods to account for endogeneity, we have estimated the association between agricultural production diversity and women’s dietary diversity among smallholder farming households in the Sidi Bouzid governorate (central Tunisia). Although we found a low level of agricultural production diversity and a fairly diversified diet among women, we observed a systematic weak positive association between five different indicators of agricultural production diversity and women’s dietary diversity. We observed a stronger positive association between women’s dietary diversity and women being more educated and households being wealthier. Neither diversity of food supplies in food markets nor market distance were associated with women’s dietary diversity, whereas we observed a higher level of consumption of some products (dairy) when they were produced on the farm.

## Introduction

Investing in the agricultural sector is considered a key strategy for eradicating poverty, hunger and malnutrition, particularly in rural areas where agriculture provides the main source of food and income [1]. Many conceptual frameworks have been developed to analyze the pathways between agriculture and food security in rural smallholder farming households [2] and among these pathways, two have been widely studied: the improvement of household food security through (i) the consumption of own food production (subsistence pathway), and (ii) income from the sale of food produced (income pathway). In the context of studies on the effects of agricultural production diversity, there are debates in the scientific community as to the level of diversification appropriate for improving dietary diversity. While numerous empirical studies have found a positive effect of production diversity on the diversity of diets [3,4], some authors have found that higher production diversity at the farm level reduces household income due to foregone earnings from specialization [5]. In Indonesia, however, increased specialization during the period 2000–2015 was associated with higher income but lower dietary diversity [6]. Furthermore, regional market food availability and accessibility can affect the links between production diversity and dietary diversity [7–9]. These observations often depend on the context of the study, and more specifically on the local food supply and whether smallholder production is more subsistence-oriented or market-oriented. In addition, these observations also depend on how the endogeneity of the relationship between production diversity and dietary diversity, observed when markets are imperfect or incomplete, have been addressed by the authors.

In Tunisia, agriculture is both a strategic sector for the economy, with the potential for creating jobs and boosting rural development, and a critical pillar of its food sovereignty [10]. However, the governorate of Sidi Bouzid, cradle of the Jasmine Revolution, is a predominantly agricultural territory that had not benefited from the economic growth and investments made in the country at the end of the 1980s [11]. The level of poverty is high, with poor access to jobs and high job insecurity, especially for women [12]. Despite the semi-arid climate, agriculture in the area has taken advantage of the fertile plains to diversify its production and benefits from large reserves of underground water. But a strong urban demand for water [13] has aggravated the pressure on water resources leading to a reduction in irrigated areas [14].

Studies over the past decade have explored the relationships between diversity in the agricultural production of rural farming households and the dietary diversity of household members [3,4], mostly using data from sub-Saharan African, and to the best of our knowledge, this question has never been explored in the Maghreb region. Using a dataset collected as part of the MEDINA study [15] in the Sidi Bouzid governorate from November 2014 to January 2016, the aim of this study was to estimate the association between annual agricultural production diversity and women’s annual dietary diversity. Focusing on women’s diet is especially important given that women are particularly vulnerable to nutritional deficiencies and women’s social status, control over resources, and health are central to many of the pathways linking agriculture and nutrition [16].

## Material and methods

### Setting and study design

The Sidi Bouzid governorate is situated in central Tunisia and has a predominantly rural population (429,912 inhabitants in 2014, of whom 73% are rural) [17]. While the area is characterized by a semi-arid climate and modest rainfall concentrated during the months of September to May [18], it constitutes one of the main Tunisian agricultural regions due to favorable underground water resources. The agricultural landscape of Sidi Bouzid shifted from traditional extensive pastoralism in the 1960s to a more productive mixed agro-pastoral system. The number of large farms deep-drilling into aquifers has increased, creating fierce competition for access to water resources between these few large farms and traditional smallholder agriculture, which is still predominant in the region [19]. The Sidi Bouzid governorate is currently one of the main contributors to the Tunisian agricultural production system, with a large diversity of production: arboriculture with olive and almond production, vegetable production, cereal, meat and more recently dairy production [20]. With a high and rising unemployment rate in the governorate, agriculture is a fallback sector for employment, especially for women [17]. Similar to the change in the agricultural landscape, the food landscape has shifted from households relying on their own production (subsistence farming) to households relying increasingly on markets for their food provision [21]. Overall, the Tunisian population is affected by a typical nutrition transition, featuring a high prevalence of excess adiposity and an increase in consumption of both ‘healthy’ and ‘unhealthy’ foods [21–23]. Compared to the national picture, central Tunisia seems to be less advanced in the nutrition transition with a lower prevalence of obesity (20.1% vs 26.2% at national level) and diabetes (10.8% vs 15.5% at national level) among adults [24].

We use data from a two-stage survey carried out as part of the MEDINA study [15]. The first stage of the survey focused on individual food intake of women aged 20–49 years, randomly selected by a cluster sampling procedure which has been described in full elsewhere [25]. Women were interviewed four times at three-monthly intervals, from November 2014 to October 2015. Based on the same sample, the second stage of the survey focused on women living in agricultural households and they were interviewed from December 2015 to January 2016 about their household and farm characteristics [26]. From these two stages resulted a sample of 304 women. While the sample was designed to be representative of women in the governorate, the random sub-sample of women living on farms can be considered to cover the heterogeneity of family farming in Sidi-Bouzid. Farms with an entrepreneurial model, where the labor force does not come from the family, were not included in this study.

### Data collection and management

#### Dietary data

Women’s food intakes were assessed by a 24-hour recall. Dietary recalls were conducted by trained personnel who asked the women to list the foods and beverages consumed during the 24 hours preceding the interview. Detailed recipes of food preparations consumed by the women were also collected. During baseline interviews, GPS coordinates, data on the relationship to the head of the household, sex and physiological status, age, marital status, professional occupation and level of education of each household member were recorded. Housing characteristics and ownership of appliances were also recorded in order to compute an asset-based wealth index through principal components analysis [27].

#### Farm data

Women were interviewed about their domestic and agricultural activities, own income and participation and decision-making in household spending during the previous year. Thereafter, the head of each farm was interviewed about crop and livestock species grown or raised during the 2015 agricultural year and whether production was destined for sale, for donation or for own consumption. Production was estimated in monetary value. First the head of the farm reported what proportion of their production was sold and the price at which it was sold. Then, production destined for donation or for own consumption was valued using the same selling prices. In cases where the product selling price was not available, an average price was calculated based on prices declared by the other farmers in the survey. The head of each farm was also interviewed about farm infrastructure (irrigation, for example), equipment and workforce (family and/or salaried labor force). Data were collected by trained personnel using standardized measurement protocols and questionnaires.

### Data management

#### Data from the MEDINA study

Quality checks were used during data entry, and double entry was performed with EpiData software version 3.1 (EpiData Association, Odense, Denmark, 2008). After the exclusion of women who did not answer at least three interviews in the dietary survey (n = 3) and exclusion of households engaged in agricultural activities but without any production during the past year (n = 11), 290 women/households/farms with complete information were available for analysis.

#### Secondary data on food markets

Data on food availability in the weekly food markets of the Sidi Bouzid governorate were extracted from the study by Jellali [28]. Briefly, 24 food markets out of the governorate’s 31 were visited from February 2016 to April 2016 to collect data about availability of food in each market. Some 1,652 vendors were interviewed about the type of food sold, prices and variety or breed. The GPS coordinates of each market were used to assign a virtual market to each household: it corresponded to the two markets with the shortest distance as the crow flies to the household and this assignment was based on the hypothesis of exclusive frequentation of the closest markets.

### Calculation of diversity indicators

#### Dietary diversity

A dietary diversity score was calculated for each woman as a proxy of the nutrient adequacy of their diet [29]. While individual dietary diversity is generally positively associated with nutrient adequacy, it does not reflect all dimensions of diet quality [30,31]. Based on each 24-hour recall administered every three months, seasonal food intakes of each woman were classified according to the 10 food group classification of the Minimum Dietary Diversity for Women (MDD-W) indicator: (1) Grains, white roots and tubers, and plantains; (2) Pulses; (3) Nuts and seeds; (4) Dairy; (5) Meat, poultry and fish; (6) Eggs; (7) Dark green leafy vegetables; (8) Other vitamin A-rich fruits and vegetables; (9) Other vegetables and (10) Other fruits [28]. Olive oil consumption is not taken into account in the calculation because it does not provide any essential micronutrients [32] although it has been demonstrated to be beneficial for human health [33]. No limit on the minimal consumption of a food group was applied. A score, the WDDS-10, was computed by counting the number of food groups consumed. Seasonal WDDS-10 scores were computed as well as an annual WDDS-10, calculated as the mean of the four seasonal scores. Achieving minimum diet diversity is defined as consuming foods from five or more food groups.

In order to understand the relationship between producing and consuming a food group, we calculated an annual consumption score for each of the 10 food groups of the WDDS-10 and olive oil. The values of this score are 0, 0.25, 0.5, 0.75 or 1 for each woman depending on whether she consumed this group in none, one, two, three or four of the 24-hour recall administered every three months.

#### Agricultural production diversity

Bogard *et al*. have highlighted the importance of using a set of indicators of production diversity for a comprehensive evaluation of the nutrition sensitivity of food production systems [34], so we calculated five different agricultural production diversity scores for each farm.

The first agricultural production diversity score was a simple sum of the number of different crop or animal products produced by each farm. A total of 29 different crop species or animal products were listed: oat, barley, wheat, potato; bean; almond; milk (regardless of the species); meat of beef, camel, goat, sheep, poultry; egg; chard; carrots, parsley; cucumber, fennel, onion, pea, squash, tomato; grape, lemon; garlic, honey, hot pepper, olive oil and table olive. The crop species or animal product was coded as 1 if the farm reported producing it and 0 otherwise. The crop species or animal products were summed into a score named Production Diversity Index (PDI).

The second agricultural production diversity score was based on the principle of the Simpson index. The crop or animal products produced by each farm were weighted by their relative abundance. Because the estimation of production during the 2015 agricultural year was in monetary value, the relative abundance of each crop species or animal product was estimated as a monetary value. This score is designated as the Simpson Diversity Index (SDI) and calculated as follows:

$$SDI=1-\;\sum_{i=1}^{n}s_{i}^{2}$$ (1)

where *s*_*i*_ is the share of the value of i^th^ crop or animal production.

The third agricultural production diversity score was a simple sum of the number of different groups of products produced by each farm. The groups were the same as those of the 10 food group classifications used in the MDD-W indicator. A group was coded as 1 if the farm reported producing it and 0 otherwise. The groups were summed into a score designated as the Group Production Diversity Index (GPDI), ranging from 0 to 10. The GPDI did not take into account some food products (garlic, hot pepper and olive oil, for example).

The fourth agricultural production diversity score was based on the principle of the Simpson index. The groups of products, based on the 10 food group classifications used in the MDD-W, produced by each farm were weighted according to their relative abundance. The relative abundance of each group was estimated as the sum of monetary value of each crop or animal product constituting the group divided by the total monetary value of the 10 groups. This score was named the Group Simpson Diversity Index (GSDI). The computation was the same as that mentioned in Eq (1).

The fifth agricultural production diversity score was based on the version of the Nutritional Functional Diversity (NFD) proposed by Luckett et al. [35]. This score was calculated following to four steps. The first step consisted in creating a product–nutrient matrix where each row was a crop or animal product found in the farm survey and each of the columns was a nutrient. This matrix was composed of 18 nutrients: protein, vitamin A, vitamin E, thiamin, riboflavin, niacin, pantothenic acid, folate, vitamin B12, vitamin C, calcium, copper, iron, magnesium, potassium, sodium and zinc. Nutritional values of the Tunisian food composition table were used [36], supplemented by the US Department of Agriculture table [37], additional laboratory analyses and the Food Processor software, version 8.3 [38]. The nutrient values in the matrix were standardized by being divided by the WHO nutrient recommendations for adult women of reproductive age [39], and then standardized to have mean = 0 and SD = 1. The second step consisted in converting the product–nutrient matrix into a product–product distance matrix, by calculating the Euclidean distance between each product using PROC DISTANCE from the SAS statistical software (version 9.4). The third step consisted in producing a cluster diagram (dendrogram) based on the distance matrix using SAS’s PROC CLUSTER and PROC TREE. The final steps consisted in using the dendrogram to calculate the NFD score. The potential NFD is calculated by summing all the branch lengths of the dendrogram and the NFD of each farm is then calculated as a percentage of this potential NFD, ranging from 0 to 100.

The five indicators of agricultural production diversity are differently related to the subsistence and income pathways and how they can affect dietary diversity. Hypothetically, SDI and GSDI could be more sensitive to income pathways, while PDI, FGPI and NFD could be more sensitive to subsistence pathways. In addition, PDI, FGPI and NFD could be considered as being differently associated with dietary diversity. The PDI counts the different crop or animal products produced by each farm regardless of their nutritional composition, which could lead to an over-contribution of products with very similar nutritional composition (wheat, barley and oat, for example) to the score [40]. The FGPI overcomes this issue by considering 10 groups of crop species or animal products but does not take into account some food products (garlic, hot pepper and olive oil, for example), while the NFD takes into account all food products and their differences in nutritional composition. Nevertheless, these three indicators fail to consider the amount of different food productions, unlike the SDI and GSDI.

#### Market diversity

A market diversity index (MDI) was available in the study of Jellali [28] and used in our analysis. Briefly, the MDI was calculated for each of the 24 markets to provide a measure of the biodiversity of available foods, based on the principle of the Shannon index and calculated as follows:

$$MDI=-\;\sum_{i=1}^{n}P_{i}\;\text{ln}\;P_{i}$$ (2)

where *P*_*i*_ is the proportion of the i^th^ crop or animal species sold in each market.

Each household was associated with a MDI calculated as the mean of the market diversity scores from the two markets closest to the home as the crow flies, resulting in a Household Access to Market Diversity Indicator (HAMDI). We used the HAMDI as a proxy of the diversity of food supplies that can be theoretically accessed by each household. The distance to the nearest market was calculated from the Euclidean distance between the GPS points locating the farms and the GPS points locating the two closest open-air markets.

### Empirical model

In this study, we were interested in determining the influence of agricultural production diversity on women’s dietary diversity. In the context of developing countries, rural households are systematically exposed to market imperfections and constraints, referred to as “market failures”. While the food supply market in Sidi Bouzid seems to be efficient, households in the governorate have been exposed to other market failures, especially the employment market. In terms of model specifications, the presence of market failure leads to what has been called non-separability [41,42].

The empirical specification can be motivated by a household model with a connection between diversity of agricultural production and household consumption. Considering a household that derives its utility from consumption (***C***) and leisure (***L***), the household utility function can be specified as ***U***(***C***, ***L***). The household is assumed to maximize utility subject to the constraints imposed by production, total household time endowment and household income. Consumption is the sum of consumption of own production and of purchased market goods. Leisure is dependent on family farm labor, family off-farm labor and, indirectly, dependent on labor hired on-farm. Consumption is constrained by budget, which is the sum of farm profits, off-farm income and savings and can be modeled with the following equation:

$$C=f\;(P\;\left(X,K,T\right),\;I,M,Y)$$ (3)

where ***P*** is the farm profit which depends on ***X*** (quantity of labor), ***K*** (capital) and ***T*** (fixed assets, such as land, for example), ***I*** is other off-farm income, ***M*** is the market as a vector of food prices and input prices, and ***Y*** is the household and individual characteristics.

In our aim to estimate the role of the diversity in production on food consumption, taking account of the non-separability could improve the estimation quality. Consumption and production are therefore observed with regard to their diversity. Market prices of inputs and food goods are considered homogeneous and only observed with regard to territorial heterogeneity measured by the type of geographical area. The market is observed with regard to the diversity of its supply measured by the market diversity scores.

First, to analyze the relationship between agricultural production diversity and dietary diversity, we use the following set of regression models:

$$y=\beta_{0}+\beta_{1}A+\;\varepsilon$$ (4)

where y denotes the women’s annual dietary diversity (annual WDDS-10) and the variable A is one of the 5 measures of production diversity (PDI, SDI, GPDI, GSDI or NFD). The constant term is β_0_, the coefficient to be estimated is β_1_ and ε is the error term.

In considering the empirical model described in Eq (3), we extend the regression models (4) as follows:

$$y=\beta_{0}+\beta_{1}A+\;\beta_{2}B+\;\beta_{3}C+\;\beta_{4}D+\varepsilon$$ (5)

where y still denotes the women’s annual dietary diversity (annual WDDS-10) and the variable A is one of the 5 measures of production diversity (PDI, SDI, GPDI, GSDI or NFD). The models was adjusted for a set of variables related to the women characteristics (B: age and level of education, responsibility for household expenditure, domestic work-time, presence of on-farm activities, presence of off-farm agricultural income, and presence of non-agricultural income), the household characteristics (C: size of the household, age of the head of the household, and wealth score) and the food supply characteristics (D: diversity of food supplies that can be theoretically accessed by each household (HAMDI) and distance from the two closest open-air markets. The constant term is β_0_, the coefficients to be estimated are β_1_, β_2_, β_3_, and β_4_, and the error term is ε.

Due to the different scale of the five production diversity indicators, the comparison of the five coefficients $\hat{\beta_{1}}$ is difficult. To overcome this issue and better understand the magnitude of the effects, the standardized $\hat{\beta_{1}}$ coefficients were also calculated as follows:

$$\hat{\beta_{1}}std=\hat{\beta_{1}}*\;\frac{S_{X}}{S_{Y}}$$ (6)

where *S*_*X*_ *and S*_*Y*_ are respectively, estimate standard errors of x et y.

Household and individual dietary diversity are influenced by a multitude of factors, some of which may not be observed or fully captured in our dataset. Under the hypothesis of non-separability, there are unobserved factors that jointly affect production diversity and dietary diversity (such as ability or behavior of farmers). This endogeneity problem may lead to a biased estimate when using the OLS model. Several authors have addressed the problem of endogeneity in estimating the links between agricultural production diversity and dietary diversity by using instrumental variable (IV) strategies [7,43–45]. Our instrumental variable strategies are based on the characteristics of the governorate of Sidi Bouzid which are exogenous factors to the farmers’ decision.

**Fig 1:**
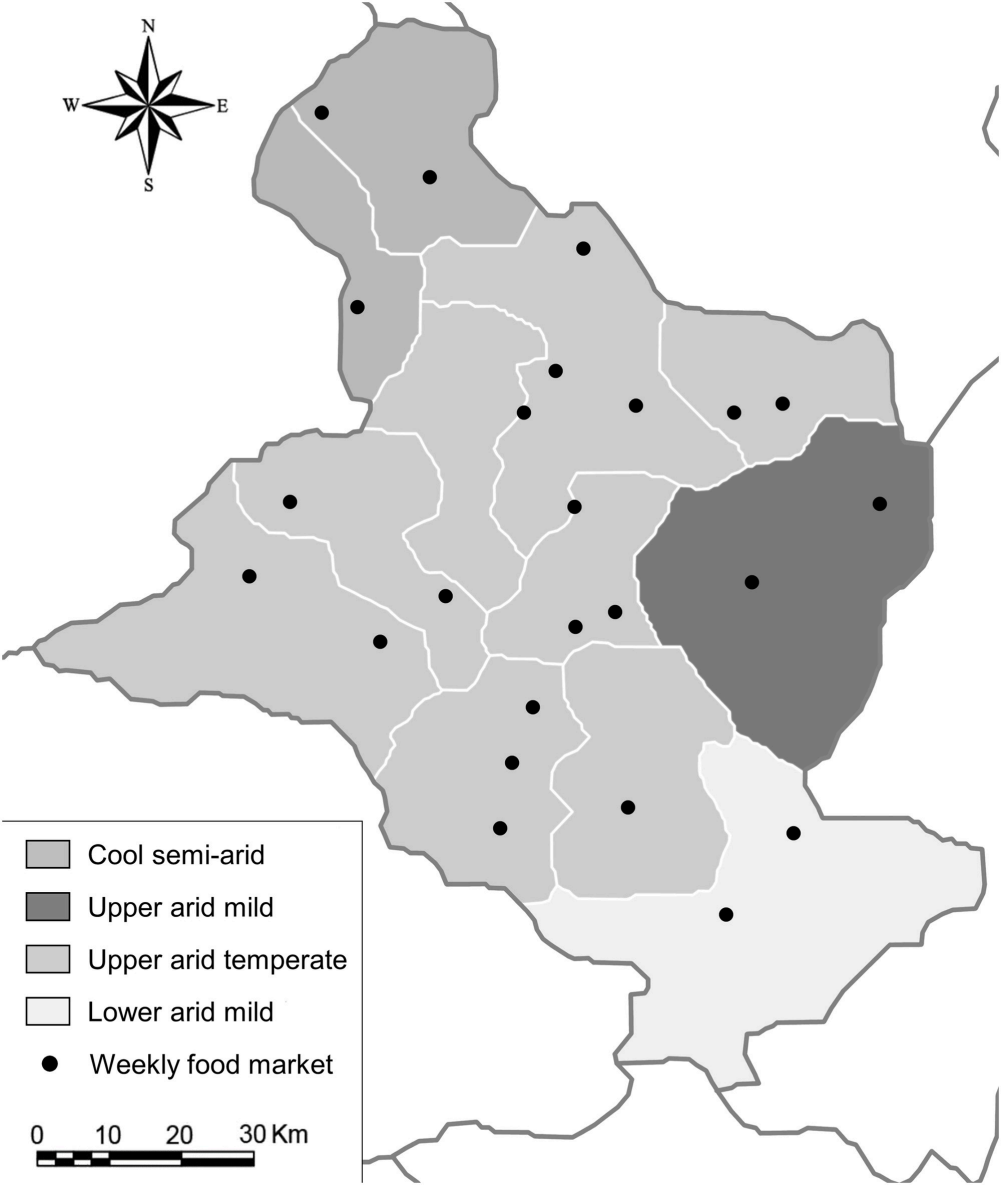
Map of the governorate of Sidi Bouzid. Main agro-climatic conditions of each of the 12 delegations are represented by 4 gray shades (upper arid mild in the east–darker gray; upper arid temperate in most of the delegations–light gray; cool semi-arid in the north–dark gray; lower arid mild in the south–lighter gray) and 24 food markets are marked as square symbols. Map modified for illustrative purposes from maps available on https://upload.wikimedia.org/wikipedia/commons/6/6e/Tunisia_delegations.png.

First, farmers’ production choices are constrained by the agro-climatic conditions in which they are located. While a major part of the governorate of Sidi Bouzid is located in a temperate semi-arid climatic zone, differences are apparent [46] with a cooler climate in the north, a drier climate in the south and a milder arid climate in the east (Fig 1). These differences could greatly influence access to water resources, topography and soil quality, and thus could influence the use of agricultural land and diversity of production. However, geographical location could also influence food accessibility and dietary diversity. To ensure that the climatic zone is only indirectly related to dietary diversity through its influence on production diversity, we controlled our model for distance to the closer markets and diversity of the surrounding markets.

Secondly, we have considered the seniority of the farm as a pertinent instrumental variable. This variable corresponds to the duration of the farmer’s use of the land. The agricultural history of Sidi Bouzid shows a strong reduction of the surface area per farm and a transition from an extensive pastoral system to a land occupation more focused on cereal and olive cultivation. Farmers who obtained their land through inheritance most often have benefited of herds from their ancestors, while new farmers have turned to more specialized systems. In addition, greater experience could be linked to a greater ability to diversify these agricultural skills and facilitate agricultural diversification strategies. To prevent the potential effects of capital accumulation over time and of a generational effect that could be related to dietary diversity, we controlled our model for household wealth and age of the head of the farm.

The limited-information maximum likelihood estimator (LIML) [47] predates the two-stage least squares (2SLS) estimator. Unlike 2SLS, the LIML estimator is invariant to the normalization used in a simultaneous equations system. Moreover, LIML and 2SLS are asymptotically equivalent given homoscedastic errors. The LIML estimator is obtained by joint ML estimation of the single Eq (5) plus the reduced form for the endogenous regressors in the right-hand side (the production diversity score) assuming heteroscedastic normal errors corrected with the Huber-White Sandwich Estimator [48]. LIML is more robust when we suspect weak instruments.


$$A=\gamma_{0}+\sum_{1}^{k}\gamma_{k}Z_{k}+\mu$$ (7)

With each ***Z***_*k*_ orthogonal with (ε,μ) and jointly normal with covariance matrix Σ.


$$\Sigma =\text{E}\left[\left(\left(\begin{array}{c} \varepsilon \\ \mu \end{array}\right){\left(\begin{array}{c} \varepsilon \\ \mu \end{array}\right)}^{\prime }\right)\right]$$ (8)

To assess the validity of the instruments, diagnostic tests were carried out. First, weak testing of the instruments developed by Stock and Yogo [49] was carried out to verify if excluded instruments are correlated with endogenous regressors, but only weakly. Estimators may perform poorly when the instruments are weak, but LIML is much more robust than the various estimators with weak instruments. Second, the Sargan–Hansen test of overidentifying restriction was performed to test if the instruments were uncorrelated with the error term, and that the excluded instruments are correctly excluded from the estimated equation. If rejected, the instrumental variable approach is not validated.

### Ethical considerations

The objective of the MEDINA study was explained to the participants prior to data collection and all participants included in the study gave their free and informed consent. The study was approved by the Tunisian National Statistics Council (visa number: 08/2014).

## Results

### Descriptive analysis

Table 1 provides summary statistics for women’s, household and farm characteristics. Our final sample included 290 women, the majority of whom had a level of education lower than middle school and reported having no professional occupation. While almost all women were involved in housework and in farm work, a minority had personal income from off-farm agricultural activities or from non-agricultural activities.

**Table 1:** Summary statistics of women’s, household and farm characteristics (n = 290)^1^.

	Mean (SD) or Median (Q1-Q3)	%
**Women’s characteristics**		
Age (years)	35.0 (7.9)	
BMI (kg/m^2^)	25.7 (5.0)	
< 18.5		5.9
18.5–24.99		43.5
25–29.99		30.3
≥ 30		20.3
Dependent child	1 (0–2)	
Domestic work-time (hours/week)	25.3 (18.5–32)	
Level of education		
No educational background		23.1
Primary school		34.8
Middle school		31.0
High school and university		11.1
Responsibility for household expenditure		4.1
Participation in on-farm activities		85.5
Presence of agricultural income (off-farm)		22.8
Presence of non-farm income		9.3
Annual WDDS-10	6.53 (1.05)	
MDD-W (≥5)		92.4
**Household characteristics**		
Age of head of household (years)	44.6 (12.3)	
Household size	5.31 (1.81)	
Wealth index	50.4 (44.3–59.8)	
Non-agricultural household income (USD)	245 (0–441)	
**Farm characteristics**		
Seniority of the farm (years)	25 (15–35)	
PDI	3 (2–4)	
SDI	0.28 (0–0.49)	
GPDI^2^	2 (1–2)	
GSDI^2^	0 (0–0.27)	
NFD	19.4 (14.9–23.8)	
Production of specified food group		
Grains, white roots and tubers		6.2
Pulses		2.1
Nuts and seeds		6.9
Dairy		19.7
Meat and poultry		81.4
Eggs		31.7
Dark green leafy vegetables		0.7
Vitamin A-rich fruits and vegetables		3.8
Other vegetables		11.4
Other fruits		0.7
Olive oil		53.5
Farm size (hectares)	2 (1–4)	
Value of production (USD/year)	1,960 (702–5,946)	
Value of production kept for own consumption (USD/year)	349 (132–817)	
Share of value of production kept for own consumption	20.6 (4.6–54.9)	
**Market characteristics**		
HAMDI	1.48 (1.41–1.54)	
Distance to closer market (km)	11.04 (6.06–14.35)	

^1^N = 290.

WDDS-10 = Women Dietary Diversity Score; MDD-W = Minimum Dietary Diversity for Women; PDI = Production Diversity Index; SDI = Simpson Diversity Index; GPDI = Group Production Diversity Index; GSDI = Group Simpson Diversity Index; NFD = Nutritional Functional Diversity; HAMDI = Household Access to Market Diversity Indicator. ^2^ The production of olive oil is not taken into account in the calculation of the FGPI and GSDI.

The 290 households of the women interviewed had an average size of 5.31 (range from 1 to 11, SD = 1.81). While all the households were engaged in agricultural activities, most of them had additional income from non-agricultural activities. Households had potential access to markets providing a large and relatively uniform diversity of food groups across the 24 markets of the Sidi Bouzid governorate.

The agricultural production diversity was relatively low in the area, with a median value of three different crops or animal products produced per farm. Among agricultural activities, raising livestock was the most common activity, followed by olive oil production. The median value of annual production was 1,960 USD, with a factor of about 50 between the 10th and 90th percentile. The share of the value of production kept for own consumption followed a U-shaped distribution, with 48.3% of the households keeping less than 20% of the value of annual production for self-consumption while 20.7% kept more than 80% for self-consumption.

#### Seasonal differences in dietary diversity

The women’s average diet was well diversified with more than six different food groups being consumed and more than 90% of the women achieved a minimum dietary diversity over the year (Table 1). While the mean seasonal WDDS-10 significantly varied across seasons, the magnitude of the variation was small. The mean WDDS-10 was the highest during spring (mean = 6.70, SD = 1.41) compared to summer (mean = 6.42, SD = 1.48), autumn (mean = 6.45, SD = 1.41) and winter (mean = 6.59, SD = 1.55). Only the differences between spring and autumn and between spring and summer were statistically significant at the 1% level. The percentage of women not reaching the minimum dietary diversity of five food groups did not vary across seasons.

**Fig 2:**
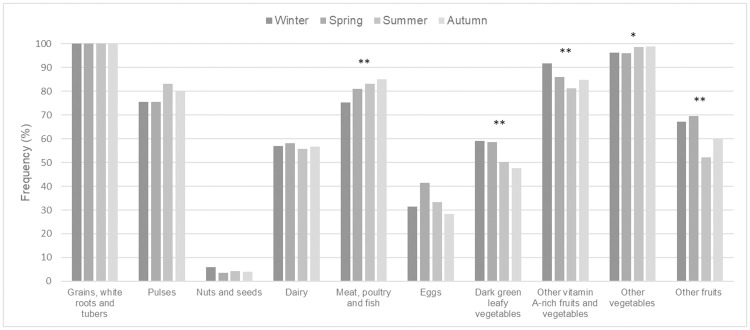
Percentage of women consuming specific food groups across four seasons in the Sidi Bouzid governorate (n = 290). Significance at the 5%, 1% level indicated by *, ** respectively using Cochran–Mantel–Haenszel test.

Seasonal variation in the percentage of consumers of individual food groups remained modest overall (Fig 2). Across seasons, the diet of all women was consistently based on grains, white roots and tubers. In contrast, few women regularly consumed eggs and very few consumed nuts and seeds. At least half of the women regularly consumed the other food groups. Olive oil, like other types of oils and fats, is not taken into account in the calculation of the WDDS-10 because it does not provide any essential micronutrients. Nevertheless, due to the high proportion of households producing olive oil, we specifically explored its consumption by women. Olive oil consumption was very common across the sample and throughout the year, with 93.8% of consumers during winter, 93.2% during spring and summer and 90.8% during autumn.

#### Production and consumption of specific food groups

Table 2 provides women’s annual consumption score for the 10 food groups of the WDDS-10 and olive oil according to whether or not these food groups were produced on the farm. We observed that women living on a farm producing dairy products had a significantly higher score of consumption of dairy products than women living on a farm that did not produce them (+30%). We also observed a significant difference for olive oil but the difference was much less important (+5%). There was no significant difference in consumption score of other food groups according to whether or not they were produced on the farm.

**Table 2:** Women’s annual level of consumption score of food groups according to whether or not they are produced by the farm^1^.

Production from specified food group (% of farms producing the group)	Mean annual consumption score (SD)	
	Producing	Not producing
Grains, white roots and tubers (6.2%)	0.97 (0.08)	0.97 (0.08)
Pulses (2.1%)	0.88 (0.14)	0.76 (0.23)
Nuts and seeds (6.9%)	0.06 (0.14)	0.04 (0.12)
Dairy (19.7%)	0.67 (0.29)	0.53 (0.35)**
Meat and poultry (81.4%)	0.79 (0.26)	0.80 (0.26)
Eggs (31.7%)	0.30 (0.29)	0.34 (0.28)
Dark green leafy vegetables (0.7%)	0.38 (0.18)	0.53 (0.28)
Vitamin A-rich fruits and vegetables (3.8%)	0.84 (0.20)	0.84 (0.20)
Other vegetables (11.4%)	0.95 (0.10)	0.95 (0.12)
Other fruits (0.7%)	0.88 (0.18)	0.61 (0.30)
Olive oil (53.5%)	0.92 (0.14)	0.88 (0.20)*

^1^N = 290.

The values of the annual consumption score are 0, 0.25, 0.5, 0.75 or 1 for each woman depending on whether she consumed a group in none, one, two, three or four of the 24-hour recall administered every three months. Significance at the 5%, 1% level indicated by *, ** respectively using Wilcoxon signed-rank test.

**Fig 3:**
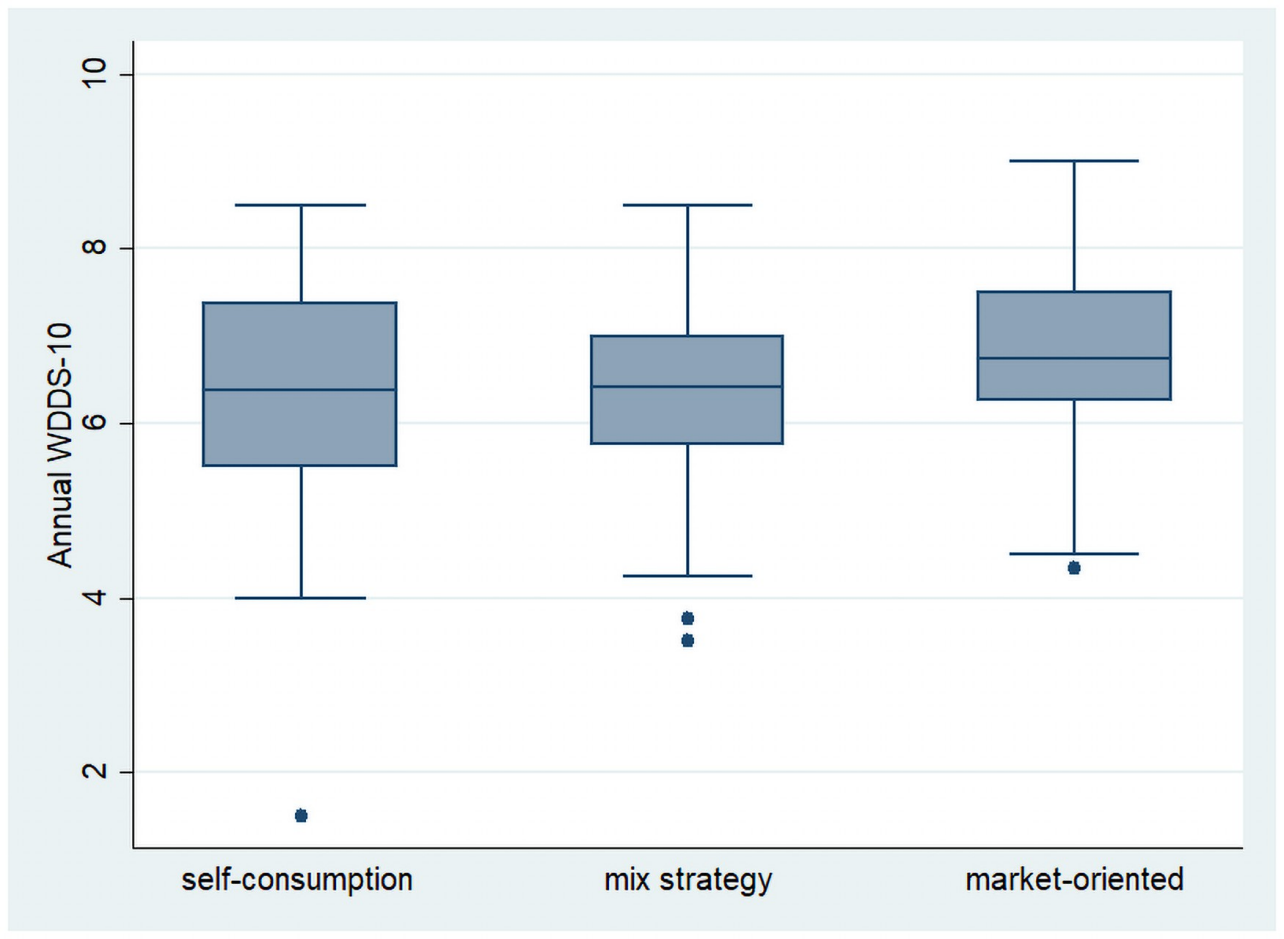
Box-plot of women’s annual dietary diversity scores (annual WDDS-10) according the orientation of agricultural production (n = 290). Self-consumption: farms where 80% of the value of annual production is kept for self-consumption (n = 60). Market-oriented: farms where 80% of the value of annual production is sold (n = 140). Mixed: the farms with a more balance distribution of the outlets of use of its production (n = 90).

Agricultural production diversity was positively correlated with the total value of production (r ranged from 0.46 to 0.53) but was not correlated with the share of value of production kept for self-consumption. Fig 3 shows the distribution of women’s annual dietary diversity scores (annual WDDS-10) according to the orientation of agricultural production of the farm. We classified the farms into three groups: the farms where more than 80% of the value of annual production is kept for self-consumption (n = 60), the farms where more than 80% of the value of annual production is sold (n = 140) and the farms with a more balance distribution of the outlets of use of its production (n = 90). We found that the dietary diversity of women living on market-oriented farms (mean = 6.74) was slightly higher than other women (mean = 6.35 in both groups, p<0.05 with a Kruskall-Wallis rank test).

### Regression results

The results of the simple regressions (Table 3) showed a positive and significant association between the five different measures of production diversity and women’s annual dietary diversity score (annual WDDS-10). However, this association was relatively weak with, for example, an increase in women’s dietary diversity of 0.077 on average for the production of additional crop species or animal products. The standardized coefficients show homogeneous effects on dietary diversity for the five agricultural production indicators: 0.127 for the PDI, 0.134 for the SDI, 0.107 for the GPDI, 0.092 for the GSDI and 0.154 for the NFD.

**Table 3:** Simple regression between agricultural production diversity and women’s dietary diversity^1^.

	Annual WDDS-10				
	PDI	SDI	GPDI	GSDI	NFD
Agricultural production diversity	0,077**	0.559**	0.108**	0.484*	0.023***
Constant	6.29***	6.38***	6.35***	6.47***	6.07***

^1^N = 290.

Significance at the 10%, 5%, 1% level indicated by *, **, ***respectively. WDDS-10 = Women Dietary Diversity Score; PDI = Production Diversity Index; SDI = Simpson Diversity Index; GPDI = Group Production Diversity Index; GSDI = Group Simpson Diversity Index; NFD = Nutritional Functional Diversity.

Table 4 provides the results of the five multiple regression models relating women’s annual dietary diversity score (annual WDDS-10) with different measures of production diversity. Diagnostic tests for the five models indicated the statistical validity of the choice of instrumental variables. First, the underidentification test and the test for weak instruments (values of Cragg-Donald Wald F superior to Stock-Yoko weak critical values of 4.84) were rejected, confirming the relevance of the instruments excepted for SDI (estimators can perform poorly in this case with maximal size estimator bias slightly higher than 10%). Second, the Sargan–Hansen test of overidentifying restriction was rejected, indicating consistent instruments (i.e. uncorrelated with the error term) and an appropriate strategy to address endogeneity.

**Table 4:** Agricultural production diversity, market access and women’s dietary diversity^1^.

	Annual WDDS-10				
Model	1	2	3	4	5
**Farm characteristics**					
Agricultural production diversity: PDI^2^	0.100*	-	-	-	-
Agricultural production diversity: SDI^2^	-	0.847	-	-	-
Agricultural production diversity: GPDI^2^	-	-	0.272*	-	-
Agricultural production diversity: GSDI^2^	-	-	-	2.081*	-
Agricultural production diversity: NFD^2^	-	-	-	-	0.024*
**Women’s characteristics**					
Age of woman	-0.005	-0.010	-0.004	-0.007	-0.005
Level of education					
No educational background (reference)	-	-	-	-	-
Primary	0.331**	0.304*	0.352**	0.290*	0.321*
Secondary	0.527***	0.455**	0.590***	0.507***	0.529***
Superior	0.960***	0.891***	0.993***	0.902***	0.942***
Domestic work-time	-0.007	-0.005	-0.007	-0.005	-0.007
Responsibility for household expenditure	0.088	0.105	0.096	0.057	0.093
Presence of non-farm income	-0.216*	-0.178	-0.244*	-0.189	-0.227*
Presence of agricultural income (off-farm)	-0.097	-0.099	-0.088	-0.041	-0.095
Participation in on-farm activities	-0.019	0.010	-0.081	-0.108	-0.011
**Household characteristics**					
Age of household head	-0.007	-0.006	-0.006	-0.006	-0.007*
Size of household	0.104***	0.101***	0.104***	0.112***	0.099***
Wealth score	0.025***	0.026***	0.023***	0.022***	0.025***
**Market characteristics**					
HAMDI	0.923	1.075	0.586	0.612	0.805
Distance to closer market	-0.039	0.245	-0.183	-0.016	-0.229
Constant	4.441***	4.309***	4.859***	5.208***	4.422***
Underidentification test	p = 0.000	p = 0,005	p = 0.004	p = 0.000	p = 0.000
Weak identification test (Cragg-Donald Wald F)	19.15	4.578	5.523	5.653	10.756
Overidentifying restriction	p = 0.754	p = 0.587	p = 0.939	p = 0.929	p = 0.813

^1^N = 290.

Significance at the 10%, 5%, 1% level indicated by *, **, ***respectively. WDDS-10 = Women Dietary Diversity Score; PDI = Production Diversity Index; SDI = Simpson Diversity Index; GPDI = Group Production Diversity Index; GSDI = Group Simpson Diversity Index; NFD = Nutritional Functional Diversity; HAMDI = Household Access to Market Diversity Indicator.

After correcting for endogeneity and controlling for the effects of several covariates on women’s annual dietary diversity, all indicators remained positively and weakly associated with annual MDDW-10 (p<0.1) except for SDI (p = 0.302). The value of the standardized coefficients revealed larger differences in the strength of these associations than with the simple regressions. The standardized coefficients for the effects of the GPDI and GSDI indicators (0.270 and 0.397 respectively), which are based on the 10 food group classifications, were higher than for the PDI and NFD indicators (0.163 and 0.165 respectively). Three covariates in the five models showed consistent and significant relationships with women’s annual dietary diversity. In each model, women’s level of education and household wealth score were positively associated with annual WDDS-10 while the size of household was negatively associated. For example, a woman with a superior education had an average annual dietary diversity that was about one food group higher (range of values from 0.891 to 0.993) than a woman with no educational background, all other covariates being equal. There was a significant negative relationship between women’s annual dietary diversity and women having personal income from non-agricultural activities (not significant in the models using SDI and GSDI). There were neither a significant relationship between women’s annual dietary diversity and the diversity of food supplies that can be theoretically accessed by each household (HAMDI), nor between women’s annual dietary diversity and distance to closer markets.

## Discussion

On one side, we found that agricultural production diversity was low among smallholder farming households in central Tunisia whose main agricultural activities are livestock and olive oil production. On the other side, we found that women living in agricultural households had a fairly diversified diet over the year. Despite this situation, we found a systematic positive but weak association between five different indicators of agricultural production diversity and dietary diversity among women living in farm households, regardless of the diversity of food supplies in food markets or market distance. To the best of our knowledge, this question has never been explored in the Maghreb region.

### Production and dietary diversity indicators

While our results are consistent with numerous studies that in different contexts have found an overall weak yet positive association between agricultural production diversity and women’s dietary diversity as measured by the WDDS-10 [9,43,49–54], methodological differences have to be highlighted.

First, as mentioned by Harris-Fry et al. [52], a temporal mismatch between agricultural production and diet assessment could affect the association between these two variables. Many studies were affected by a temporal mismatch with a reference period of 24-hour for dietary data and a reference period for agricultural production of one year [50,52,53] or one season [9,41]. In addition, previous studies have shown that women’s dietary diversity is sensitive to seasonality [55,56]. Like Lourme-Ruiz *et al*. [54], our study avoided this pitfall, as we relied on 24-hour recalls administered every three months to calculate an annual dietary diversity score which enabled us to partially overcome the temporal mismatch.

Second, the assessment of the capacity of a production system to improve the diet is influenced by the choice of indicator of production diversity [34] but most studies are based on only one [9,50–52] or two [43,53] indicators. For example, Lourme-Ruiz *et al*. used four indicators of agricultural production diversity in the context of a cotton-growing region in rural western Burkina Faso, and found that only one indicator was associated with women’s dietary diversity [54]. In our study, we found that all of the five different indicators were associated with women’s dietary diversity.

Third, agricultural production diversity in our study (median of two different groups of crops and animal products) was one of the lowest observed in the literature, together with Bellows *et al*. who found an average farm diversity of 1.62 crop groups [51], compared to the agricultural production diversity observed in other studies. For example, Jones *et al*. [53], Adubra *et al*. [50] and Harris-Fry *et al*. [52] found an average diversity of respectively 5.8, 4.2 and 3.6 different crop and animal species or products.

Finally, Tunisia is considered less vulnerable to food insecurity compared to most of the countries where relationships between agricultural production diversity and women’s dietary diversity have been evaluated [57]. In our study, WDDS-10 was much higher (mean = 6.53) compared to other studies (median or mean varying from 3 to 4.8). Minimum dietary diversity was achieved by more than 90% of the women in our study, while at best 55% achieved it in the Peruvian Andes [53], falling to less than 6% in rural Ethiopia [9].

### Pathways between production and dietary diversity

In contrast to a significant share of the literature exploring the relationship between agricultural production diversity and dietary diversity, our study did not take place in a context where farmers give priority to the production of staple crops that are the basis of the local diet [58]. In the Sidi Bouzid governorate, the availability of cereal products on the market (e.g. couscous) is mainly ensured by imports and national production [59], and to a lesser extent by a few local farms with entrepreneurial management (not included in our sample). In our study, farmers are mainly engaged in raising livestock and producing olive (largely sold for olive oil export). So, among the pathways through which agricultural can impact nutrition [60], the income pathway (income from agriculture that can be used to purchase diverse food) might be expected to have a strong and positive effect on women’s dietary diversity. In descriptive analysis, we found that agricultural production diversity was positively correlated with the total value of production, and that women living in market-oriented farms had slightly higher dietary diversity score. However, agriculture as a source of food for self-consumption should not be overlooked. We observed a higher consumption of dairy products in women living on a farm which produced dairy products compared to women on non-producing farms. Similarly, women on olive-growing farms had a slightly higher consumption of olive oil. While olive oil consumption is not taken into account in the calculation of dietary diversity, consuming it from their own production could be associated with savings in the food budget which might be reallocated to purchase other food products and thus increase dietary diversity. Indeed, olive oil consumption is relatively weak in Tunisia due to its high price in comparison to other vegetable oils [61] especially imported soybean oil [62].

It might be in the interest of public policies to promote the better valuing of olive production at the local level, which has a beneficial effect on the income of more than half of local family farmers, and thereby a potential positive indirect influence on the diversity of their diet. The under-exploitation of the sector in the country has been highlighted, in particular caused by inadequate storage and transport technologies [63]. But olive crops constitute high-value products and favor the development of local agro-processing industries. Olive production has a clear comparative advantage in Tunisia, and in Sidi Bouzid in particular. While olive production has demonstrated good resistance to hydric stress and a great capacity to adapt to different climates [64], public policies should encourage agro-ecological practices associated with better water management in an area suffering from water scarcity. It also might be of interest to support the dairy sector that remains particularly fragile with many informal actors downstream of dairy production, and suffers from a lack of incentives for innovation [20]. The poor availability of green fodder and forage areas is a limiting factor for dairy production in Sidi Bouzid. Animal feed is supplied on a fodder market run mainly by the owners of the milk collection centers, who speculate on prices and limit the profit margin of breeders [65].

There was no significant relationship between women’s dietary diversity and diversity of food supplies or distance to closer market. In a context where the food supply is important, accessible and diverse [66], it can be expected that controlling for these variables does not alter the relationship between production diversity and women’s dietary diversity. Nevertheless, our analysis suffers from limitations regarding this aspect. The MDI was initially calculated by Jellali [28] to provide a measure of the biodiversity of foods in weekly food markets of the Sidi Bouzid governorate. We used this indicator as a proxy of the food supplies that can be theoretically accessed by households, knowing that these markets represent only a fraction of the local food environment. Although it was mainly documented in urban areas, Tunisian households have frequent recourse to small neighborhood grocery stores for food purchases [67].

### Women’s characteristics, production and dietary diversity

In our study, women’s individual factors were diversely associated with their dietary diversity. The most robust one was the level of education, contributing significantly and positively to greater dietary diversity. This result has already been observed in Tunisia [68], but also in other contexts like the Peruvian Andes [53] and rural Mali [50]. However, unlike Komatsu *et al*. [69], we found that women’s involvement in domestic work, including meal preparation, was not significantly associated with a decrease in dietary diversity.

We found a negative effect of participation in non-farm activities, which nevertheless improves women’s livelihoods and so promotes their economic access to a range of products. As already observed in the region, the more family members are involved in on-farm activities, the better the women’s food security. Reciprocally, it was observed that too much time spent on extra-agricultural activities is linked to a decrease in dietary diversity. However, with regards to obtaining non-farm income, it should be noted that it is closely linked to educational level and contributes to the level of household wealth.

Faced with the combination of a tight labor market and unfavorable social and cultural norms [70] or the requirement for mobility through internal or external migration to gain access to employment [71], the empowerment of women remains a major issue in Tunisia. This is particularly the case in rural areas, where a study has highlighted the low level of empowerment of Tunisian women in comparison with levels of empowerment of women in Bangladesh, Guatemala and Uganda, despite Tunisia having a higher overall level of economic and human development [72]. Nevertheless, initiatives are undertaken within the framework of the Tunisian Strategic Plan for Nutrition (2018–2022) with interventions supported by the World Food Programme for women living in rural areas aiming to empower them by producing healthy and nutritious food for local markets [73].

## Conclusion

The connection between agriculture and food is a crucial issue in the Sidi-Bouzid governorate, where the reproductive capacity of a large proportion of small-scale family farmers was considerably reduced by the economic crisis preceding the social crisis of the Arab Spring. Our results suggest that agricultural production diversity was low among smallholder farming households in central Tunisia and not oriented towards production of staple crops, while women living in these households had a fairly diversified diet, indicating a diversified and accessible food market supply at the regional level. Despite this situation, we found a systematic positive but weak association between five different indicators of agricultural production diversity and dietary diversity among women living in farm households. Our results suggest that this relative benefit on women’s dietary diversity came through the income pathway but also the subsistence pathway (particularly striking for dairy consumption). The problems of precariousness and access to employment, which persist in rural Tunisia, make public interventions necessary. These policies must both target the region’s dominant agricultural sectors, such as olive production, to improve household livelihoods and promote diversified agriculture that is more resilient to the food price fluctuations that have contributed to the impoverishment of local populations.
